# Bridging Guidelines and Oral Healthcare: Development and Initial Validation of a Questionnaire Assessing Early-Career Dentists’ Attitudes Toward Periodontal Therapy—A Cross-Sectional Study

**DOI:** 10.3390/healthcare14131938

**Published:** 2026-07-01

**Authors:** Cristian Cojocaru, Dragos Ioan Virvescu, Dana Gabriela Budala, Monica Silvia Tatarciuc, Florinel Cosmin Bida, Gabriel Rotundu, Oana-Maria Butnaru, Florin Razvan Curca, Teona Ana-Maria Tudorici, Ionut Luchian

**Affiliations:** Grigore T. Popa University of Medicine and Pharmacy, 700115 Iasi, Romania

**Keywords:** periodontal disease, periodontal therapy, surveys and questionnaires, psychometrics, clinical practice guidelines

## Abstract

**Background/Objectives**: Contemporary periodontal therapy is increasingly guided by evidence-based clinical protocols, particularly the European Federation of Periodontology (EFP) S3 Clinical Practice Guidelines. The present study aimed to assess dentists’ perceptions regarding modern periodontal therapy and to perform a preliminary psychometric evaluation of a newly developed questionnaire. **Methods**: A cross-sectional questionnaire-based study was conducted among 109 dental practitioners. The questionnaire evaluated perceptions regarding non-surgical periodontal therapy, surgical periodontal therapy, and the comparative role of both therapeutic approaches. Descriptive statistical analysis, internal consistency analysis using Cronbach’s Alpha, Intraclass Correlation Coefficient (ICC), repeated measures ANOVA, and exploratory factor analysis (Principal Component Analysis—PCA) with Varimax rotation were performed to evaluate the psychometric properties of the instrument. **Results**: The questionnaire demonstrated good internal consistency, with Cronbach’s Alpha values ranging from 0.77 to 0.86. ICC analysis confirmed satisfactory reliability. Respondents showed favorable attitudes toward evidence-based periodontal management, supporting non-surgical therapy as first-line treatment and the staged integration of surgical approaches in complex cases. Exploratory factor analysis identified a coherent factorial structure, with four components explaining 73.94% of the total variance. **Conclusions**: The questionnaire showed good psychometric properties, supporting its potential utility as a reliable and useful instrument for evaluating professional attitudes and clinical decision-making in periodontology.

## 1. Introduction

Because of their complex causes, progressive tissue damage, and lasting effects on oral health and quality of life, periodontal diseases are still an important issue in modern dental practice and one of the most common chronic inflammatory conditions affecting the oral cavity [1,2]. Treatment options vary from simple non-surgical periodontal therapy to complex surgical regenerative procedures, with the main emphasis being on controlling the microbial biofilm and modulating the host inflammatory response, taking into account the severity of the disease and its clinical presentation [3]. The significance of organized, sequential therapeutic protocols and personalized treatment planning in the management of periodontitis has been highlighted by recent developments in evidence-based periodontology and the release of the S3 Clinical Practice Guidelines of the European Federation of Periodontology (EFP) [4,5].

Regardless of the stage of condition, non-surgical periodontal therapy is still the first-line therapeutic strategy and remains the foundation of periodontal treatment. Significant clinical improvements in decreasing inflammation, probing depth, and disease progression have been achieved through procedures involving modalities such as scaling and root planing, professional plaque control, supra- and subgingival instruments, and supportive periodontal therapy [5,6]. A growing number of adjunctive technologies have recently attracted interest due to their ability to enhance clinical outcomes and patient comfort [7]. These technologies include subgingival air polishing, guided biofilm therapy (GBT), vector systems, laser-assisted therapy, and subgingival air polishing [8]. Additionally, evidence-based clinical guidelines recommend avoiding systemic antibiotics unless absolutely necessary, and this is rarely performed in high-risk cases [9].

Despite the effectiveness of conservative approaches, periodontal surgical therapy remains essential in advanced clinical situations characterized by deep periodontal pockets, vertical bone defects, furcation involvement, and limited accessibility for non-surgical instrumentation [10].

Although current guidelines clearly support a staged therapeutic approach, the decision-making process regarding the timing and indication of surgical versus non-surgical therapy may vary considerably among clinicians, depending on professional experience, postgraduate education, clinical setting, and familiarity with contemporary evidence-based recommendations [11].

The success of periodontal therapy is strongly influenced not only by the selected treatment modality but also by patient-related factors [12]. Effective daily oral hygiene practices represent a cornerstone of periodontal disease management, contributing significantly to biofilm control, reduction in inflammation, and long-term stability of clinical outcomes. Patient compliance with oral hygiene instructions has consistently been identified as one of the strongest predictors of treatment success and disease control [13].

In addition, several lifestyle-related factors may substantially influence the progression of periodontitis and the response to therapy. Smoking is recognized as one of the most important modifiable risk factors, being associated with increased disease severity, impaired healing, and less favorable treatment outcomes [14].

Dietary habits may also affect periodontal health through their influence on systemic inflammation and host immune responses [15]. Furthermore, long-term adherence to supportive periodontal care programs is essential for maintaining treatment results and reducing the risk of disease recurrence [16]. Consequently, contemporary periodontal management requires not only appropriate therapeutic interventions but also active patient participation and sustained behavioral modifications.

Although several studies have investigated dentists’ knowledge, attitudes, and clinical practices related to periodontal disease management, the available instruments have generally focused on specific aspects of periodontal care, educational outcomes, or adherence to individual treatment approaches. To the best of our knowledge, no validated questionnaire has been specifically developed to comprehensively assess clinicians’ perceptions regarding the contemporary integration of non-surgical and surgical periodontal therapies within the framework of current EFP S3 guideline recommendations.

This gap in the literature is particularly relevant given the increasing complexity of periodontal decision-making and the growing emphasis on evidence-based treatment strategies. By providing a structured and validated assessment tool, this study contributes to a better understanding of clinicians’ attitudes toward modern periodontal therapy and may support future educational, clinical, and research initiatives.

Therefore, the aim of this preliminary study was to evaluate dentists’ perceptions regarding modern non-surgical and surgical periodontal therapies, as well as the comparative relationship between these two therapeutic approaches, from the perspective of the EFP S3 Clinical Practice Guidelines. A secondary objective was to perform a preliminary psychometric evaluation of the questionnaire developed for this purpose, including the assessment of internal consistency, reliability, and factorial structure, in order to explore its potential applicability as a research instrument in future larger-scale studies.

## 2. Materials and Methods

Recent advances in evidence-based periodontology and the increasing implementation of EFP S3 Clinical Practice Guidelines have highlighted the importance of understanding clinicians’ perspectives regarding contemporary periodontal therapeutic strategies.

### 2.1. Study Design

The study was conceived as a cross-sectional observational investigation aimed at evaluating dentists’ perceptions regarding non-surgical and surgical periodontal therapy, as well as the comparative relationship between these two therapeutic approaches, from the perspective of the European Federation of Periodontology (EFP) S3 Clinical Practice Guidelines. Data collection was performed using a structured anonymous questionnaire administered exclusively for scientific research purposes. Data collection was conducted between March 2026 and May 2026 among dental practitioners practicing in Romania.

### 2.2. Study Population and Sampling

The study sample consisted of 109 dentists with different levels of professional experience and specialization. Data collection was performed electronically using the Survio^®^ online survey platform (Survio s.r.o., Brno, Czech Republic). The questionnaire link was distributed via email exclusively to dental professionals in order to reduce self-selection bias associated with unrestricted public dissemination through social media or messaging platforms. To minimize the risk of multiple submissions and ensure that each participant contributed only one response, several control measures were implemented, including restriction to one response per IP address, browser cookie activation to prevent repeated submissions from the same device, and controlled distribution of the survey link without public access.

Sample size estimation was performed according to commonly accepted recommendations for exploratory factor analysis and preliminary psychometric validation studies, which suggest recruiting between 5 and 10 participants per questionnaire item. Considering the 18 Likert-scale items included in the present questionnaire, a minimum sample ranging from 90 to 180 participants was considered methodologically acceptable. The final sample of 109 respondents met the minimum recommended threshold for exploratory psychometric evaluation and preliminary construct validation. A flow chart summarizing participant recruitment, response screening, and final sample inclusion is presented in the Results Section.

Participants were also explicitly instructed to complete the questionnaire only once. In addition, post hoc screening of the collected responses was performed to identify potential duplicate or invalid entries based on identical response patterns, identical or near-identical timestamps, and unusually short completion times. Suspected duplicate or invalid responses were excluded from the final dataset following manual verification.

Participation was voluntary and anonymous, and written informed consent was obtained electronically from all participants. Completion of the questionnaire required approximately 10–12 min. All responses were automatically recorded electronically and anonymized prior to statistical analysis.

Participants received a standardized invitation message containing a brief description of the study purpose, information regarding confidentiality and anonymity, and an estimated completion time. No incentives were offered for participation. Reminder emails were sent during the data collection period in order to improve the response rate.

All participants received identical invitation text to ensure methodological consistency and reduce the risk of information bias.

### 2.3. Questionnaire Description

The research instrument used in the present study was a questionnaire entitled “Modern Perspectives for Optimizing Diagnosis and Surgical versus Non-Surgical Therapy in Periodontology”, specifically developed for this study based on the available scientific literature and the current recommendations of the EFP S3 Clinical Practice Guidelines. The questionnaire was originally developed and administered in Romanian. The English version presented in this manuscript is a translated version provided for reporting purposes only. The questionnaire consisted of four sections:

- Section 1—demographic and professional data (6 items), including gender, specialty, professional experience, work environment, postgraduate training, and familiarity with the S3 Guidelines;
- Section 2—perception of modern non-surgical periodontal therapies (7 items);
- Section 3—perception of surgical periodontal therapy (6 items);
- Section 4—comparison between non-surgical and surgical approaches (5 items).

Items included in Sections 2–4 were evaluated using a 5-point Likert scale ranging from “strongly disagree” (score 1) to “strongly agree” (score 5). The total number of Likert-scale items was 18.

The questionnaire development process followed several sequential stages. Initially, a comprehensive review of the contemporary periodontal literature and the recommendations included in the European Federation of Periodontology (EFP) S3 Clinical Practice Guidelines was performed in order to identify the principal concepts and clinical domains relevant to modern periodontal therapy. Based on this review, an initial pool of questionnaire items was generated to cover perceptions regarding non-surgical periodontal therapy, surgical periodontal therapy, and the relationship between these therapeutic approaches.

Subsequently, the preliminary version of the questionnaire was evaluated by a panel of five academic experts with experience in periodontology, dental public health, and clinical research methodology. The experts independently assessed each item for relevance, clarity, comprehensibility, and consistency with the objectives of the study. Based on their recommendations, several items were reworded to improve clarity and reduce ambiguity, while preserving the original conceptual framework.

Content validity was assessed qualitatively through expert consensus regarding the adequacy of item coverage of the investigated constructs. Following this review process, the final version of the questionnaire was considered appropriate for administration and subsequent psychometric evaluation.

### 2.4. Statistical Analysis

Statistical analysis was performed using IBM SPSS version 29.0 Statistics software (IBM Corp., Armonk, NY, USA), and the level of statistical significance was set at *p* < 0.05.

Descriptive Analysis—For each questionnaire item, the mean, standard deviation, and frequency distribution were calculated in order to characterize response distribution patterns and the general tendency of respondents’ opinions.

Internal Consistency Analysis—The internal consistency of the questionnaire and of each individual section was assessed using Cronbach’s alpha coefficient. Values of α ≥ 0.70 were considered indicative of acceptable reliability. Item–total analysis was also performed, including corrected item–total correlations and evaluation of Cronbach’s alpha variation following the deletion of each individual item.

Intraclass Correlation Coefficient (ICC)—The stability and internal agreement of the scale were additionally evaluated using the Intraclass Correlation Coefficient (ICC), calculated for both Single Measures and Average Measures. Statistical significance was assessed using the associated F-test.

Repeated Measures ANOVA—To evaluate the discriminatory capacity of the scale, repeated measures ANOVA were performed to analyze differences between the mean scores of items within each questionnaire section. The presence of statistically significant differences was interpreted as an indicator of the instrument’s sensitivity to variations in clinical perception.

Exploratory Factor Analysis—The adequacy of the dataset for factor analysis was assessed using the Kaiser–Meyer–Olkin (KMO) test and Bartlett’s test of sphericity. Subsequently, the latent structure of the questionnaire was investigated using Principal Component Analysis (PCA).

To obtain a clearer factorial structure, orthogonal rotation was applied, and communalities were analyzed in order to evaluate the contribution of each item to the extracted factors. Items with communality values ≥ 0.50 were considered adequately represented within the factorial model.

The study was conducted in accordance with the ethical principles of biomedical research and the provisions of the Declaration of Helsinki. Ethical approval was obtained from the Ethics Committee of the Grigore T. Popa University of Medicine and Pharmacy of Iași, Romania (protocol code 741 on 22 March 2026). Participation was voluntary and anonymous, and informed consent was considered implicitly obtained upon completion and submission of the questionnaire.

## 3. Results

**Figure 1:**
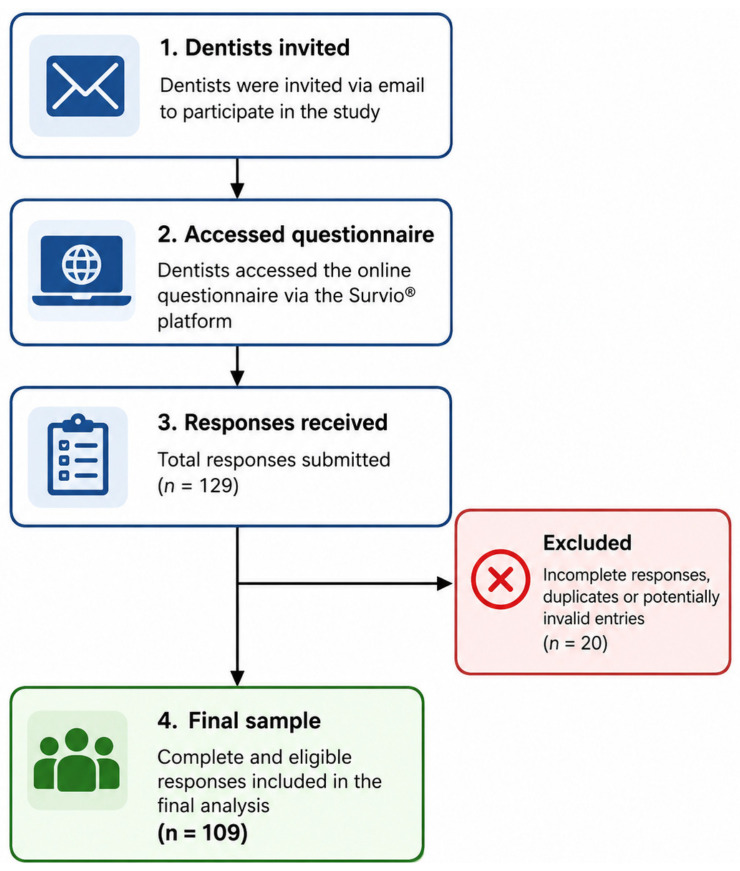
Flowchart of participant selection, response screening, and final inclusion process.

A total of 109 complete questionnaires were included in the final statistical analysis. After exclusion of 20 incomplete, duplicate, or potentially invalid questionnaires, 109 complete questionnaires were included in the final statistical analysis as seen in Figure 1. The data obtained allowed the evaluation of dentists’ perceptions regarding contemporary non-surgical and surgical periodontal therapies, as well as the psychometric assessment of the questionnaire used in the present preliminary study.

### 3.1. Section 1. General Characteristics of the Study Sample

The analysis of respondents’ distribution according to gender demonstrated a predominance of female participants within the studied sample. Thus, 67.0% of participants were female (*n* = 73), while 33.0% were male (*n* = 36). This distribution reflects the current structure of the dental professional workforce, in which female participation is considerably increased, and provides adequate representativeness for the analysis of perceptions regarding the investigated periodontal therapies.

The analysis of the sample structure according to professional specialization revealed a predominance of general dental practitioners, who represented 51.4% of respondents (*n* = 56). Periodontology residents accounted for 22.0% of the sample (*n* = 24), reflecting the increased interest of this professional category in the study topic. Participants with other dental specialties represented 23.9% of the sample (*n* = 26), providing a multidisciplinary perspective regarding the investigation of the therapeutic approaches. A smaller proportion, namely 2.8%, consisted of certified specialists or senior specialists in periodontology (*n* = 3), which may be explained by the more limited numerical distribution of this professional category. Overall, the sample structure provided adequate professional diversity relevant to evaluating perceptions regarding periodontal therapy.

The analysis of respondents’ distribution according to years of professional experience in dental practice demonstrated a marked predominance of early-career practitioners. Most participants, namely 91.7% (*n* = 100), reported less than 5 years of professional experience, while 8.3% (*n* = 9) indicated between 5 and 10 years of experience. This distribution suggests that the sample consisted predominantly of young dentists actively engaged in professional development and adaptation to contemporary clinical guidelines and practices, an aspect particularly relevant for the evaluation of perceptions regarding modern therapeutic approaches in periodontology.

The analysis of the primary professional practice setting showed that most respondents were active in the private sector. Thus, 42.2% of participants (*n* = 46) worked in multidisciplinary private clinics, while 34.9% (*n* = 38) practiced in individual dental offices. A smaller proportion, namely 11.0% (*n* = 12), worked in hospitals or university clinics, whereas 11.9% (*n* = 13) reported other forms of professional practice organization. This distribution reflects the diversity of dental practice environments and provides a relevant context for interpreting perceptions regarding the applicability of periodontal therapies across different clinical settings.

The analysis of participation in postgraduate courses or continuing education programs in periodontology during the previous five years revealed variable levels of educational involvement among respondents. More than half of the participants (54.1%, *n* = 59) reported that they had not attended such courses during the evaluated period. In contrast, 27.5% (*n* = 30) indicated occasional participation in continuing education programs, while 18.3% (*n* = 20) reported consistent participation, defined as attending at least one course per year. This distribution suggests the existence of a gap between the need for continuous professional updating and the actual level of access or interest in postgraduate training, an aspect relevant for interpreting perceptions regarding the implementation of contemporary guidelines and periodontal therapies in clinical practice.

### 3.2. Section 2. Perception of Modern Non-Surgical Periodontal Therapies

The section evaluating the perception of modern non-surgical periodontal therapies demonstrated good internal consistency, with Cronbach’s alpha coefficient of 0.86 across the 7 analyzed items, indicating satisfactory reliability of the research instrument as illustrated in Table 1.

Descriptive analysis revealed generally high levels of agreement across all items, with mean scores ranging from 3.89 to 4.11 in Table 2. The highest level of agreement was observed for Item 11, while the lowest mean score was recorded for Item 7.

The Item–Total statistics analysis for the section evaluating the perception of modern non-surgical periodontal therapies is presented in Table A1 and demonstrated an adequate contribution of all items to the internal consistency of the scale. Corrected item–total correlation values ranged between 0.49 and 0.75, exceeding the minimum acceptable threshold of 0.30, indicating that each item was relevant and appropriately correlated with the total scale score.

Cronbach’s alpha values if item deleted ranged between 0.82 and 0.87. The removal of none of the items resulted in a substantial increase in the alpha coefficient compared with the initial scale value (α = 0.86), confirming that all items were appropriately integrated and necessary for maintaining the reliability of the instrument.

Item–total analysis supported the retention of all seven items within the questionnaire structure, as all corrected item–total correlations exceeded the recommended threshold and no item deletion improved the overall reliability of the scale.

The psychometric evaluation of the scale dedicated to the perception of modern non-surgical periodontal therapies as seen in Table A2 demonstrated good reliability, with a Cronbach’s alpha coefficient of 0.86, supporting the internal consistency of the instrument. Item–total correlation analysis further supported the retention of all seven items within the scale.

Repeated measures ANOVA revealed statistically significant differences between items (F = 2.62; *p* = 0.01), indicating variability in responses across the evaluated aspects of non-surgical periodontal therapy in Table A2. The overall mean score was 4.001, reflecting a generally positive perception of the concepts investigated.

The Intraclass Correlation Coefficient (ICC) analysis for the scale evaluating the perception of modern non-surgical periodontal therapies, presented in Table A3, demonstrated good measurement reliability, particularly when the average item score was used.

The ICC for Single Measures was 0.46 (95% CI: 0.38–0.55), indicating moderate agreement, whereas the ICC for Average Measures reached 0.86 (95% CI: 0.81–0.89), indicating high reliability of the overall scale. The associated F-test was statistically significant (F = 7.13; *p* < 0.001), supporting the consistency of the measurements. These findings further support the reliability of the instrument for assessing perceptions regarding modern non-surgical periodontal therapies.

### 3.3. Section 3. Perception of Surgical Periodontal Therapy

The internal consistency of the scale evaluating the perception of surgical periodontal therapy was assessed using Cronbach’s alpha coefficient. As presented in Table 3, the six-item scale demonstrated good internal consistency, with Cronbach’s alpha value of 0.78.

The descriptive statistical analysis of the six items included in the section dedicated to the perception of surgical periodontal therapy, presented in Table 4, indicated a high level of agreement among respondents, with mean values ranging between 3.90 and 4.23 in a sample of 109 participants.

Respondents generally expressed positive perceptions regarding periodontal surgical therapy. The highest levels of agreement were observed for items related to the management of vertical bone defects, deep periodontal pockets, and the use of minimally invasive surgical approaches. Overall, mean scores indicate favorable attitudes toward the application of surgical therapy in accordance with contemporary clinical guidelines.

The Item–Total statistics analysis for the section dedicated to the perception of surgical periodontal therapy, presented in Table A4 and Table A5, indicated an adequate contribution of most items to the internal consistency of the scale. Corrected item–total correlation values ranged from 0.36 to 0.69, all exceeding the recommended threshold of 0.30, indicating an adequate contribution of each item to the overall scale.

The item addressing the application of surgical therapy only after failure of non-surgical treatment, according to the S3 Guidelines, demonstrated the lowest item–total correlation; however, the value remained acceptable and may reflect variability in clinical opinion among respondents. Cronbach’s alpha values if item deleted ranged between 0.72 and 0.82, and the removal of none of the items resulted in a substantial improvement of the global alpha coefficient (α = 0.78). This finding supports the retention of all items within the scale structure and confirms its conceptual homogeneity and psychometric stability.

The ANOVA analysis demonstrated statistically significant differences between the items of the scale evaluating perceptions regarding surgical periodontal therapy (F = 6.36; *p* < 0.001), indicating that respondents evaluated specific aspects of this therapeutic approach differently. Nevertheless, the high overall mean value (Grand Mean = 4.10) reflects a generally favorable attitude toward periodontal surgical therapy. These findings confirm the ability of the scale to discriminate between different clinical dimensions while maintaining the coherence and psychometric relevance of the instrument, as illustrated in Table A6.

The Intraclass Correlation Coefficient (ICC) analysis for the section evaluating perceptions regarding surgical periodontal therapy, presented in Table A7, demonstrated acceptable to good reliability of the scale, particularly when the average item score was used. For Single Measures, the ICC value was 0.38 (95% CI: 0.30–0.47), indicating a moderate level of agreement between items, which reflects the diversity of clinical opinions regarding different aspects of surgical periodontal therapy.

In contrast, for Average Measures, the ICC reached a value of 0.78 (95% CI: 0.72–0.84), corresponding to a good level of reliability. The associated F-test was statistically significant (F = 4.72; *p* < 0.001), confirming the presence of genuine agreement between items and valid differentiation between respondents. These findings are consistent with the Cronbach’s alpha coefficient obtained for this section and support the use of the scale as a composite instrument for evaluating perceptions regarding surgical periodontal therapy. Overall, the ICC analysis confirmed that the scale demonstrates adequate psychometric stability and is suitable for further statistical analyses in clinical research.

### 3.4. Section 4. Comparison Between Non-Surgical and Surgical Approaches

The section dedicated to the comparison between non-surgical and surgical approaches demonstrated good internal consistency, with Cronbach’s alpha coefficient of 0.77 for the 5 analyzed items, as illustrated in Table 5.

The descriptive statistical analysis of the five items included in the section dedicated to the comparison between non-surgical and surgical approaches, presented in Table 6, revealed a high level of agreement among respondents, with mean values ranging between 3.71 and 4.43 in a sample of 109 participants. The highest level of agreement was observed for the statement regarding the staged integration of the two therapeutic approaches in complex cases, suggesting a clear orientation toward combined and personalized treatment strategies. In addition, non-surgical treatment was perceived as being better accepted by patients compared with surgical therapy, highlighting the importance of patient compliance and comfort in therapeutic decision-making.

Descriptive analysis indicated generally positive perceptions regarding the relationship between non-surgical and surgical periodontal therapies. Greater variability was observed for items addressing the comparability of clinical outcomes and the timing of surgical intervention, whereas stronger agreement was recorded for the role of non-surgical therapy and supportive periodontal care in preventing the need for surgery.

The Item–Total statistics analysis for the section dedicated to the comparison between non-surgical and surgical approaches, presented in Table A8, demonstrated an adequate contribution of all five items to the internal consistency of the scale. Corrected item–total correlation values ranged between 0.41 and 0.64, exceeding the minimum acceptable threshold of 0.30, thereby confirming that each item was relevant and appropriately correlated with the overall scale score.

Corrected item–total correlation values supported the contribution of all items to the overall scale. Cronbach’s alpha if item deleted ranged from 0.69 to 0.77, indicating that the removal of any individual item did not substantially improve the reliability of the instrument. Therefore, all items were retained within the final scale structure.

Repeated measures ANOVA revealed statistically significant differences between items (F = 38.12; *p* < 0.001), indicating variability in responses across the evaluated statements. The overall mean score was 4.08, reflecting generally positive perceptions regarding the integration of non-surgical and surgical periodontal therapies.

The Intraclass Correlation Coefficient (ICC) analysis for the section comparing non-surgical and surgical approaches, presented in Table A9, demonstrated good reliability of the scale, particularly when the composite score was used. The ICC value for Single Measures indicated a moderate level of agreement between items, reflecting the variety of aspects evaluated, whereas the ICC for Average Measures reached a value of 0.77, corresponding to a good level of reliability. The associated F-test was statistically significant (*p* < 0.001), confirming measurement stability and genuine differentiation between respondents. These findings are consistent with the Cronbach’s alpha coefficient and support the use of the scale as a reliable psychometric instrument for evaluating comparative perceptions regarding the two therapeutic approaches.

The results of the KMO and Bartlett’s tests, presented in Table 7, indicated that the dataset was appropriate for factor analysis. The Kaiser–Meyer–Olkin (KMO) value of 0.77 suggested a good level of sampling adequacy, indicating sufficient correlations between items for the identification of latent factors. At the same time, Bartlett’s test of sphericity was statistically significant (χ^2^ = 693.63; df = 78; *p* < 0.001), confirming that the correlation matrix differed significantly from an identity matrix. Overall, these findings support the suitability of conducting exploratory factor analysis and indicate a coherent latent structure among the analyzed items.

The communality values obtained through exploratory factor analysis (PCA), presented in Table 8, indicated an adequate representation of all items within the factorial structure, with extraction values ranging between 0.65 and 0.80. These findings demonstrate that a substantial proportion of the variance of each item was explained by the extracted factors, exceeding the minimum acceptable threshold for inclusion in the model.

The items related to the use of modern technologies, the acceptability of non-surgical treatment, the staged integration of therapeutic approaches, and the advantages of periodontal surgery demonstrated particularly high communalities, indicating strong coherence with the latent structure of the evaluated construct. Overall, the results support the retention of all items in the analysis and confirm the construct validity and adequacy of the instrument used.

The analysis of total variance explained indicated that, following exploratory factor analysis using the Principal Component Analysis (PCA) method, four components with eigenvalues greater than 1 were identified, supporting their retention within the final factorial structure. Together, these four components explained 73.94% of the total variance of the dataset, representing a high percentage and indicating a good capacity of the factorial model to capture the information contained within the questionnaire items.

The first component explained 34.97% of the total variance and demonstrated a dominant contribution to the structure of the instrument, suggesting the presence of a central dimension well represented by a coherent set of items. The second component contributed 17.61% of the variance, while the third explained 13.42%, indicating additional conceptually distinct and clinically relevant dimensions that substantially complemented the questionnaire structure. The fourth component accounted for 7.94% of the total variance and reflected an additional dimension with a clearly defined role, although with a lower contribution compared with the first three components.

Following Varimax rotation in Table 9, the explained variance became more evenly redistributed across the final components without modifying the cumulative percentage of explained variance. Thus, the first rotated component explained 22.64% of the variance, the second 19.01%, the third 18.53%, and the fourth 13.77%, indicating a more balanced distribution of information among factors. This redistribution confirms the role of rotation in clarifying the factorial structure and facilitating the conceptual interpretation of each dimension.

Components with eigenvalues below 1 were not retained, as their individual contribution to variance explanation was limited and did not provide a meaningful additional contribution to the factorial structure of the model. Overall, the high percentage of variance explained by the four retained components supports the adequacy of the factorial solution and indicates that the questionnaire is well structured, effectively capturing the main dimensions of the investigated clinical attitudes and practices.

**Figure 2:**
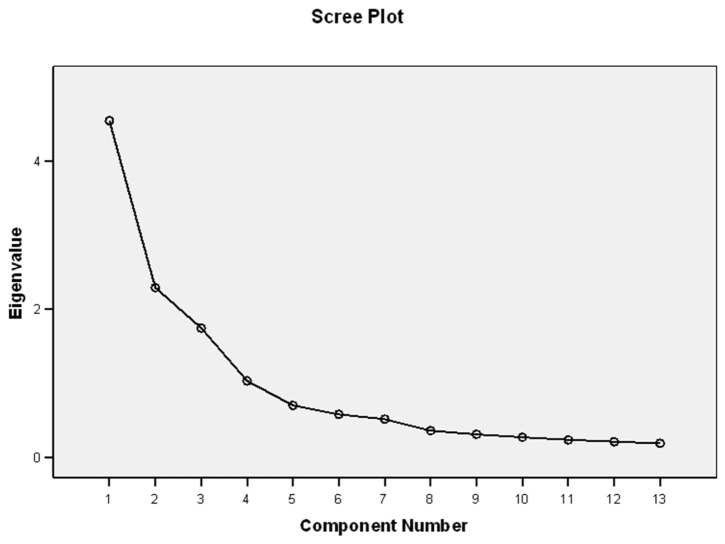
Determination of the number of questionnaire factors based on scree plot analysis.

The scree plot analysis performed for the questionnaire items, presented in Figure 2, revealed a clear inflection point after the third component, followed by a gradual decrease in eigenvalues. However, according to the Kaiser criterion, four components presented eigenvalues greater than 1 (4.54, 2.28, 1.74, and 1.03), together explaining 73.94% of the total variance presented in Table 9. Although visual inspection of the scree plot suggested a possible three-factor solution, the fourth component also met the retention criterion and contributed additional explanatory value. Therefore, a four-factor solution was retained and further explored through Varimax rotation. The resulting structure demonstrated satisfactory interpretability and conceptual coherence, supporting the retention of four factors in the final model.

The unrotated component matrix, presented in Table 10, revealed factor loadings distributed across multiple components, with numerous cross-loadings between items, indicating an insufficiently differentiated factorial structure at this stage of the analysis. This type of distribution is characteristic of the pre-rotation phase of exploratory factor analysis and does not allow a clear conceptual interpretation of the factors. Therefore, Varimax rotation was applied in order to obtain a clearer factorial structure and facilitate the interpretation of the latent dimensions.

The application of Varimax rotation with Kaiser normalization clarified the initial factorial structure and resulted in the identification of four distinct components reflecting conceptually relevant dimensions of periodontal clinical management, as illustrated in Table 11. The distribution of items across components demonstrated coherent organization, characterized by predominantly high factor loadings and minimal overlap between factors, thereby facilitating conceptual interpretation and supporting the construct validity of the instrument.

The first component grouped items related to the effectiveness and optimization of non-surgical periodontal treatment, including the use of local antiseptics, modern adjunctive technologies, rigorous application of the subgingival instrumentation phase according to the S3 Guidelines, and the judicious administration of systemic antibiotics in selected cases. The high factor loadings associated with these items indicate that respondents perceive modern guideline-based non-surgical treatment as an effective and predictable therapeutic strategy.

The second component reflected the role of periodontal surgical therapy and the criteria supporting its clinical indication. The items defining this factor addressed the preference for minimally invasive approaches, the advantages of surgical intervention in areas inaccessible to non-surgical therapy, and the necessity of surgery in the management of vertical bone defects or deep periodontal pockets. This structure suggests a differentiated understanding of surgical indications based on case selection and the clinical benefits of intervention.

The third component grouped items expressing a patient-centered conservative approach and a staged therapeutic strategy. This dimension included the perception of greater patient acceptability of non-surgical treatment, the importance of maintenance therapy and rigorous reevaluation in preventing the need for surgical intervention, and the progressive integration of non-surgical and surgical therapies in complex cases. This factor reflects a pragmatic clinical approach focused on balancing therapeutic effectiveness with patient comfort.

The fourth component captured the relationship between clinical practice and the recommendations of the S3 Guidelines, highlighting both the perception of comparable outcomes between non-surgical and surgical therapies in moderate forms of periodontitis and the tendency toward earlier use of surgery in routine clinical practice than recommended by current guidelines. This component reveals the existence of a discrepancy between theoretical recommendations and their practical implementation, emphasizing decision-making variability according to clinical context and professional experience.

Overall, the obtained factorial structure demonstrates that the questionnaire items are organized into distinct and conceptually well-defined dimensions reflecting essential aspects of periodontal decision-making. The high factor loadings and coherent distribution of items across factors support the methodological adequacy of the exploratory factor analysis and confirm that the instrument is capable of validly and distinctly capturing the investigated clinical attitudes and practices.

The component transformation matrix describes the manner in which the initial components were reoriented through the application of Varimax rotation with Kaiser normalization in order to obtain a clearer and more easily interpretable factorial structure. The presented values represent the transformation coefficients between the initial and rotated components, indicating the contribution of each initial component to the definition of the final factors.

The distribution of coefficients demonstrates that each rotated factor resulted from a linear combination of multiple initial components, without a single initial component exclusively dominating the final structure. This redistribution of variance is characteristic of orthogonal rotations and confirms that the Varimax rotation maximized relevant factor loadings while minimizing overlap between latent dimensions.

The presence of both positive and negative coefficients with varying magnitudes reflects the reorientation of the factorial axes so that each factor captures as accurately as possible a distinct conceptual dimension of the questionnaire. Furthermore, the orthogonal nature of the rotation indicates that the final factors are independent from one another, supporting their interpretation as separate dimensions of periodontal decision-making.

Within the context of the present questionnaire, this matrix confirms that the final factorial structure is not the result of arbitrary organization, but rather of a controlled statistical transformation intended to better reflect the actual relationships between items. Thus, the transformation matrix supports the stability and robustness of the factorial solution and reinforces the construct validity of the instrument, demonstrating that the identified dimensions are well differentiated and appropriate for evaluating the investigated clinical attitudes and practices (Table 12).

**Figure 3:**
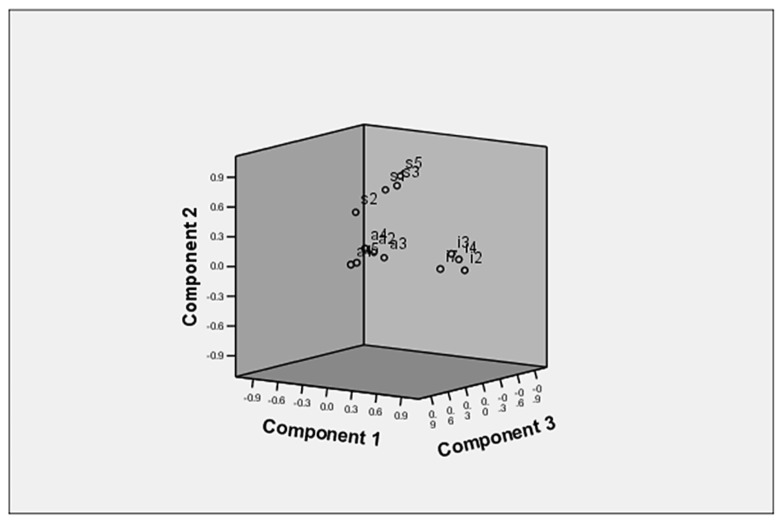
Component plot in rotated space showing the distribution of questionnaire items across the extracted factors.

The graphical representation of the items in the rotated component space, presented in Figure 3, demonstrated a coherent organization of the items around the axes corresponding to the extracted components, thereby confirming the factorial structure identified through exploratory factor analysis. Following the application of Varimax rotation, the items tended to form distinct clusters, with a clear visual separation between latent dimensions, indicating a substantial reduction in overlap between factors.

Items positioned in close proximity to the same axis represented questions measuring the same clinical construct and were perceived by respondents as belonging to the same decisional dimension. The clusters observed in the graph reflected a clear differentiation between attitudes toward non-surgical periodontal treatment, the indications and role of surgical therapy, and the alignment of clinical practice with treatment guideline recommendations. This distribution suggests that respondents consistently distinguished between conservative therapeutic strategies, surgical interventions, and protocol-guided clinical approaches.

Items located closer to the center of the graph indicated dimensions with a more general or transversal character, potentially influencing multiple aspects of clinical decision-making without being exclusively associated with a single factor. Nevertheless, these items did not compromise the overall separation of the components but rather reflected the complexity of decision-making in periodontology.

Overall, the Component Plot in rotated space provides additional visual support for the validation of the final factorial structure, demonstrating that the questionnaire items are well anchored within distinct and coherent dimensions. This organization confirms that the instrument is capable of distinctly capturing the investigated clinical attitudes and practices, thereby supporting the construct validity and practical usefulness of the questionnaire.

## 4. Discussion

The present preliminary study evaluated dentists’ perceptions regarding contemporary non-surgical and surgical periodontal therapies in relation to the EFP S3 Clinical Practice Guidelines, while also performing a psychometric assessment of the developed questionnaire. The obtained results demonstrated generally favorable attitudes toward evidence-based periodontal management and supported the reliability and construct validity of the research instrument.

One of the most important findings of the study was the high level of agreement regarding the role of non-surgical periodontal therapy as the cornerstone of periodontal treatment. Respondents strongly supported the concept that scaling and root planning, professional plaque control, and rigorous subgingival instrumentation remain fundamental therapeutic procedures, consistent with current EFP S3 recommendations. These findings are in line with the contemporary periodontal literature, which emphasizes that effective biofilm disruption and supportive periodontal therapy represent the essential basis for long-term disease control, regardless of disease stage [17,18,19].

The favorable perception regarding adjunctive technologies such as laser-assisted therapy, subgingival air-polishing, Vector systems, and Guided Biofilm Therapy (GBT) reflects the increasing integration of minimally invasive and technology-assisted protocols into contemporary clinical practice [20,21]. Although scientific literature reports promising clinical benefits for these approaches, including improved patient comfort and biofilm management efficiency, evidence regarding their superiority over conventional therapy remains heterogeneous [22,23]. Therefore, the positive perception observed among respondents may also reflect the growing influence of modern clinical trends and continuing education programs in periodontology.

An important aspect highlighted by the results was the favorable perception regarding restrictive systemic antibiotic use. Most respondents agreed that systemic antibiotics should be reserved for severe or carefully selected cases, which aligns with current recommendations regarding antimicrobial stewardship and the need to reduce unnecessary antibiotic exposure in periodontal therapy [24,25]. This finding may indicate increasing awareness among clinicians regarding evidence-based pharmacological management and the global concern related to antimicrobial resistance.

Regarding surgical periodontal therapy, respondents generally recognized its importance in advanced periodontal destruction, particularly in cases involving deep periodontal pockets, vertical bone defects, and areas with limited accessibility for non-surgical instrumentation. At the same time, the relatively great variability observed for some surgical-related items suggests that therapeutic decision-making remains influenced by clinical experience, personal therapeutic philosophy, and variability in interpretation of guideline recommendations. This aspect is particularly visible in the responses regarding the timing of surgical intervention, where some clinicians appeared to support earlier surgical approaches than those generally recommended by the S3 Guidelines.

The generally positive perceptions observed in the present study are consistent with previous questionnaire-based investigations reporting favorable attitudes among dental professionals toward evidence-based periodontal concepts and contemporary treatment recommendations. Previous studies have shown that dentists and dental trainees generally recognize the importance of guideline-based periodontal management; however, variability may still exist regarding the implementation of specific therapeutic decisions in daily clinical practice [26].

The generally positive perceptions observed in the present study are consistent with previous investigations evaluating periodontal knowledge and attitudes among dental professionals. Mohammadi-Moghaddam et al. [26] reported favorable levels of knowledge and attitudes regarding periodontal health among practicing dentists, suggesting an increasing awareness of evidence-based periodontal management.

The high level of agreement regarding non-surgical periodontal therapy, supportive periodontal care, and the staged integration of treatment approaches observed in our study may reflect the increasing emphasis placed on evidence-based periodontal education during undergraduate and early professional training. Similar findings have been reported in studies evaluating clinicians’ perceptions of periodontal treatment principles and interdisciplinary decision-making [27]. Some variability was observed regarding the timing of surgical intervention and the interpretation of specific therapeutic indications. Similar discrepancies have been reported by Lane et al. [27] who identified differences in periodontal diagnosis and treatment planning among dental students from different institutions. Such findings suggest that, despite the existence of standardized classifications and treatment guidelines, clinical decision-making may still be influenced by differences in educational experiences, clinical exposure, and individual interpretation of complex periodontal cases.

Nevertheless, the variability identified for certain items related to surgical intervention may indicate differences in clinical exposure and confidence when managing more complex periodontal cases. Such discrepancies between theoretical recommendations and real-world decision-making have also been reported in previous questionnaire-based studies among dental professionals [28].

Similarly, Yadadi et al. [28]. demonstrated a high level of awareness and acceptance of the 2017 World Workshop Classification among dental students, reflecting the growing incorporation of contemporary periodontal concepts into dental education. The favorable perceptions identified in the present study may therefore reflect the influence of modern educational curricula and the increasing dissemination of evidence-based clinical guidelines.

The consistency between the present findings and previous studies supports the growing acceptance of contemporary periodontal treatment concepts. At the same time, the observed differences highlight the need for continuous professional education and further standardization of periodontal decision-making in accordance with current evidence-based recommendations.

The present findings also have relevant clinical and educational implications. The generally favorable attitudes toward guideline-based non-surgical periodontal therapy suggest that early-career dentists are receptive to evidence-based concepts and may benefit from structured educational strategies that reinforce the staged periodontal treatment approach. In undergraduate dental education, these results support the need to integrate clinical practice guidelines more explicitly into periodontology teaching, not only as theoretical content but also through case-based learning, treatment planning exercises, and simulation of clinical decision-making.

The variability observed in responses related to surgical timing and the comparative role of non-surgical and surgical therapy indicates that some aspects of periodontal decision-making remain more difficult to standardize. This finding is particularly important for dental curricula, as students and recent graduates may require greater exposure to complex periodontal cases, interdisciplinary treatment planning, and critical appraisal of guideline-based indications. Therefore, the questionnaire developed in the present study could be used as an educational assessment tool to identify areas in which learners or early-career clinicians require additional training.

From the perspective of continuing professional development, the results highlight the importance of targeted courses focusing on the practical implementation of the EFP S3 Guidelines, patient compliance, supportive periodontal care, and the selection criteria for surgical intervention. Such programs could help reduce discrepancies between theoretical recommendations and real-world clinical decision-making, particularly among clinicians with limited experience in advanced periodontal therapy. In this context, the questionnaire may serve not only as a research instrument but also as a practical tool for evaluating training needs and monitoring the impact of educational interventions over time.

However, several limitations of the present study should be acknowledged. First, the study included a relatively limited sample size and should therefore be considered a preliminary investigation. Second, the respondent population consisted predominantly of young clinicians with less than five years of professional experience, which may have influenced the observed perceptions and may limit the generalizability of the findings to more experienced practitioners. Third, the study evaluated self-reported perceptions rather than actual clinical behavior, and therefore the results may not fully reflect real-world therapeutic decision-making. In addition, because participation was voluntary and questionnaire-based, the possibility of response bias and social desirability bias cannot be excluded.

A limitation of the present study is represented by the relatively modest sample size used for psychometric evaluation. Although the number of participants was sufficient for the exploratory analyses performed, larger samples could provide greater stability of the factorial structure and facilitate more extensive validation procedures.

In addition, all participants were recruited from the same national population, which may restrict the generalizability of the findings to other healthcare systems, educational environments, and cultural contexts. Future multicenter studies involving more diverse populations are needed to further confirm the applicability of the questionnaire across different professional settings.

Another limitation that should be acknowledged is that the study sample was predominantly composed of dentists with less than five years of professional experience. Although this characteristic reflects the target population included in the present investigation, future studies involving participants with a broader range of professional experience may provide additional evidence regarding the applicability of the questionnaire across different professional groups.

The interpretation of the present findings should also consider certain methodological limitations. Although reminder emails were sent during the data collection period to encourage participation, the exact number of dentists who received or viewed the invitation was not systematically recorded. Consequently, a precise response rate could not be calculated. This limitation restricts the ability to fully assess the representativeness of the study sample and raises the possibility of self-selection bias, as participation was voluntary and may have attracted individuals with a greater interest in periodontal therapy and evidence-based clinical practice. Therefore, the results should be interpreted with appropriate caution when extrapolating the findings to the broader population of dental practitioners.

Although the present findings support the reliability and validity of the proposed instrument, the results should be considered as an initial psychometric evaluation, and further studies are required to confirm the factorial structure and applicability of the questionnaire in broader populations.

Additional psychometric investigations, including confirmatory factor analysis, test–retest reliability assessment, and external validation on larger and more heterogeneous populations, are necessary before the instrument can be considered fully validated for extensive clinical research use.

## 5. Conclusions

The present study demonstrated that dentists generally show favorable attitudes toward evidence-based periodontal therapy, with strong support for non-surgical treatment as the foundation of periodontal management and for the selective use of surgical interventions in advanced cases. Respondents also emphasized the importance of staged therapeutic approaches, patient compliance, and adherence to the EFP S3 Clinical Practice Guidelines.

From a psychometric perspective, the developed questionnaire demonstrated good internal consistency, satisfactory reliability, and a coherent factorial structure, supporting its preliminary validity as an instrument for evaluating professional perceptions regarding periodontal therapy.

Although additional validation on larger and more heterogeneous populations is necessary, the obtained results suggest that the questionnaire may represent a valuable and useful research tool for future studies investigating clinical attitudes and guideline implementation in periodontology.

## Figures and Tables

**Table 1:** Internal Consistency of Section 2 Evaluating the Perception of Modern Non-Surgical Periodontal Therapies.

Cronbach’s Alpha	N of Items
0.86	7

**Table 2:** Descriptive Analysis of the Perception of Non-Surgical Periodontal Therapies.

Item	Mean	Standard Deviation	N
7. Standard non-surgical procedures (SRP, supragingival and subgingival scaling) are sufficient for most Stage I–II periodontal patients.	3.88	0.68	109
8. The use of local antiseptics improves the outcomes of non-surgical treatment.	4.09	0.71	109
9. Systemic antibiotic administration (e.g., amoxicillin + metronidazole) should be reserved for severe cases, according to the S3 Guidelines.	3.89	0.70	109
10. Modern technologies (e.g., LASER, subgingival air-polishing, Vector, GBT-EMS) are useful in optimizing non-surgical outcomes.	4.02	0.68	109
11. The S3 Guidelines emphasize that non-surgical therapy remains the first-line treatment regardless of disease stage.	4.11	0.76	109
12. I consider that the effectiveness of non-surgical treatment is minimal without a high level of patient compliance.	4.02	1.08	109
13. Rigorous application of Phase 2 of the S3 Guidelines (complex subgingival instrumentation) leads to significant clinical improvement even in Stage III disease.	3.96	0.66	109

**Table 3:** Internal Consistency of the Scale Evaluating the Perception of Surgical Periodontal Therapy.

Cronbach’s Alpha	N of Items
0.78	6

**Table 4:** Respondents’ Perception Regarding the Effectiveness and Indications of Periodontal Surgical Therapy.

Item	Mean	Standard Deviation	N
14. Surgical intervention is essential in the treatment of vertical bone defects or deep periodontal pockets.	4.04	0.73	109
15. Periodontal surgery should be performed only after failure of non-surgical treatment, according to the S3 Guidelines.	3.89	1.01	109
16. I prefer minimally invasive approaches in selected and eligible cases.	4.22	0.66	109
18. I consider that regenerative surgical interventions provide additional long-term advantages compared with non-surgical therapy in Stage III–IV disease.	3.98	0.75	109
19. Periodontal surgical therapy allows better visual access and control of areas inaccessible to non-surgical therapy.	4.22	0.68	109
20. Surgical procedures have a higher clinical success rate in advanced periodontal disease cases, but require careful and objective patient selection.	4.22	0.71	109

**Table 5:** Internal Consistency of the Comparative Scale between Non-Surgical and Surgical Approaches.

Cronbach’s Alpha	N of Items
0.77	5

**Table 6:** Descriptive Analysis of Items Comparing Non-Surgical and Surgical Approaches.

Item	Mean	Standard Deviation	N
21. Non-surgical treatment is better accepted by patients compared with surgical therapy.	4.35	0.70	109
22. Clinical outcomes are comparable between non-surgical and surgical therapy in moderate forms of periodontitis, according to the S3 Guidelines.	3.74	0.73	109
23. In clinical practice, surgical approaches are sometimes used earlier than recommended by the S3 Guidelines.	3.70	0.83	109
24. Non-surgical therapy combined with rigorous reevaluation and periodic professional mechanical plaque removal can prevent the need for surgical intervention.	4.17	0.79	109
25. In complex cases, the staged integration of both approaches (non-surgical + surgical) provides the best outcomes.	4.43	0.65	109

**Table 7:** KMO and Bartlett’s Test—Data Adequacy for Factor Analysis.

Kaiser-Meyer-Olkin Measure of Sampling Adequacy.		0.77
Bartlett’s Test of Sphericity	Approx. Chi-Square	693.63
	df	78
	Sig.	0

**Table 8:** Exploratory Factor Analysis (PCA).

Item	Initial	Extraction
8. The use of local antiseptics improves the outcomes of non-surgical treatment.	1	0.76
10. Modern technologies (e.g., LASER, subgingival air-polishing, Vector, GBT-EMS) are useful in optimizing non-surgical outcomes.	1	0.80
13. Rigorous application of Phase 2 of the S3 Guidelines (complex subgingival instrumentation) leads to significant clinical improvement even in Stage III disease.	1	0.72
15. Periodontal surgery should be performed only after failure of non-surgical treatment, according to the S3 Guidelines.	1	0.71
16. I prefer minimally invasive approaches in selected and eligible cases.	1	0.76
19. Periodontal surgical therapy allows better visual access and control of areas inaccessible to non-surgical therapy.	1	0.79
21. Non-surgical treatment is better accepted by patients compared with surgical therapy.	1	0.79
22. Clinical outcomes are comparable between non-surgical and surgical therapy in moderate forms of periodontitis, according to the S3 Guidelines.	1	0.68
23. In clinical practice, surgical approaches are sometimes used earlier than recommended by the S3 Guidelines.	1	0.72
24. Non-surgical therapy combined with rigorous reevaluation and periodic professional mechanical plaque removal can prevent the need for surgical intervention.	1	0.74
25. In complex cases, the staged integration of both approaches (non-surgical + surgical) provides the best outcomes.	1	0.75
9. Systemic antibiotic administration (e.g., amoxicillin + metronidazole) should be reserved for severe cases, according to the S3 Guidelines.	1	0.65
14. Surgical intervention is essential in the treatment of vertical bone defects or deep periodontal pockets.	1	0.68

Extraction method: Principal Component Analysis.

**Table 9:** Analysis of Total Variance Explained.

Component	Initial Eigenvalues			Extraction Sums of Squared Loadings			Rotation Sums of Squared Loadings		
	Total	% of Variance	Cumulative %	Total	% of Variance	Cumulative %	Total	% of Variance	Cumulative %
1	4.54	34.97	34.97	4.54	34.97	34.97	2.94	22.63	22.63
2	2.28	17.60	52.57	2.28	17.60	52.57	2.47	19.01	41.64
3	1.74	13.42	65.99	1.74	13.42	65.99	2.40	18.52	60.17
4	1.03	7.94	73.94	1.03	7.94	73.94	1.79	13.77	73.94
5	0.70	5.39	79.33						
6	0.58	4.46	83.80						
7	0.51	3.97	87.78						
8	0.36	2.77	90.55						
9	0.31	2.40	92.96						
10	0.27	2.10	95.06						
11	0.23	1.83	96.89						
12	0.21	1.63	98.53						
13	0.19	1.46	100						

Extraction method: Principal Component Analysis.

**Table 10:** Unrotated Component Matrix.

Item	Component 1	Component 2	Component 3	Component 4
8. The use of local antiseptics improves the outcomes of non-surgical treatment.	0.44	0.75	0.04	0.003
10. Modern technologies (e.g., LASER, subgingival air-polishing, Vector, GBT-EMS) are useful in optimizing non-surgical outcomes.	0.60	0.66	0.05	0.01
13. Rigorous application of Phase 2 of the S3 Guidelines (complex subgingival instrumentation) leads to significant clinical improvement even in Stage III disease.	0.63	0.54	−0.06	−0.14
15. Periodontal surgery should be performed only after failure of non-surgical treatment, according to the S3 Guidelines.	0.31	−0.34	0.53	−0.45
16. I prefer minimally invasive approaches in selected and eligible cases.	0.64	−0.16	0.56	0.07
19. Periodontal surgical therapy allows better visual access and control of areas inaccessible to non-surgical therapy.	0.55	−0.22	0.59	0.3
21. Non-surgical treatment is better accepted by patients compared with surgical therapy.	0.66	−0.27	−0.25	−0.46
22. Clinical outcomes are comparable between non-surgical and surgical therapy in moderate forms of periodontitis, according to the S3 Guidelines.	0.50	−0.32	−0.44	0.36
23. In clinical practice, surgical approaches are sometimes used earlier than recommended by the S3 Guidelines.	0.54	−0.21	−0.48	0.38
24. Non-surgical therapy combined with rigorous reevaluation and periodic professional mechanical plaque removal can prevent the need for surgical intervention.	0.76	−0.26	−0.24	−0.16
25. In complex cases, the staged integration of both approaches (non-surgical + surgical) provides the best outcomes.	0.69	−0.27	−0.34	−0.27
9. Systemic antibiotic administration (e.g., amoxicillin + metronidazole) should be reserved for severe cases, according to the S3 Guidelines.	0.59	0.54	0.03	0.09
14. Surgical intervention is essential in the treatment of vertical bone defects or deep periodontal pockets.	0.59	−0.35	0.32	0.32

**Table 11:** Application of Varimax Rotation.

Item	Component 1	Component 2	Component 3	Component 4
8. The use of local antiseptics improves the outcomes of non-surgical treatment.	0.87	−0.002	−0.02	−0.03
10. Modern technologies (e.g., LASER, subgingival air-polishing, Vector, GBT-EMS) are useful in optimizing non-surgical outcomes.	0.88	0.12	0.09	0.04
13. Rigorous application of Phase 2 of the S3 Guidelines (complex subgingival instrumentation) leads to significant clinical improvement even in Stage III disease.	0.79	0.05	0.28	0.04
15. Periodontal surgery should be performed only after failure of non-surgical treatment, according to the S3 Guidelines.	−0.12	0.55	0.44	−0.44
16. I prefer minimally invasive approaches in selected and eligible cases.	0.21	0.81	0.20	−0.02
19. Periodontal surgical therapy allows better visual access and control of areas inaccessible to non-surgical therapy.	0.12	0.87	0.01	0.09
21. Non-surgical treatment is better accepted by patients compared with surgical therapy.	0.11	0.10	0.86	0.12
22. Clinical outcomes are comparable between non-surgical and surgical therapy in moderate forms of periodontitis, according to the S3 Guidelines.	−0.007	0.13	0.29	0.76
23. In clinical practice, surgical approaches are sometimes used earlier than recommended by the S3 Guidelines.	0.10	0.09	0.27	0.79
24. Non-surgical therapy combined with rigorous reevaluation and periodic professional mechanical plaque removal can prevent the need for surgical intervention.	0.18	0.25	0.71	0.36
25. In complex cases, the staged integration of both approaches (non-surgical + surgical) provides the best outcomes.	0.13	0.11	0.78	0.32
9. Systemic antibiotic administration (e.g., amoxicillin + metronidazole) should be reserved for severe cases, according to the S3 Guidelines.	0.77	0.16	0.07	0.13
14. Surgical intervention is essential in the treatment of vertical bone defects or deep periodontal pockets.	0.03	0.75	0.15	0.31

Extraction method: Principal Component Analysis. Rotation method: Varimax with Kaiser normalization. Rotation converged in 6 iterations.

**Table 12:** Component Transformation Matrix.

Component	1	2	3	4
1	0.53	0.50	0.57	0.35
2	0.84	−0.34	−0.35	−0.22
3	0.01	0.74	−0.30	−0.59
4	0.008	0.27	−0.67	0.68

Extraction method: Principal Component Analysis. Rotation method: Varimax with Kaiser normalization.

## Data Availability

The data presented in this study are available on request from the corresponding author The data are not publicly available due to privacy ethical restrictions.

## Abbreviations

The following abbreviations are used in this manuscript:

EFP	European Federation of Periodontology
GBT	Guided Biofilm Therapy

## Appendix A

### Appendix A.1. Questionnaire

This questionnaire aims to evaluate dentists’ perceptions regarding modern non-surgical therapies in the treatment of periodontal disease and their comparison with surgical approaches, according to the recommendations of the European Federation of Periodontology (EFP) S3 Guidelines.

Estimated duration: 10–12 min. Responses are anonymous and used exclusively for scientific purposes.

### Appendix A.2. Rating Scale

1. Strongly disagree
2. Disagree
3. Neutral/No opinion
4. Agree
5. Strongly agree

#### Appendix A.2.1. Section 1: Demographic and Professional Information

1. Gender: Female/Male/Other/Prefer not to answer
2. Specialty: General dentist/Specialist-Consultant in Periodontology/Periodontology resident/Other specialty
3. Years of experience in dental practice: Less than 5 years/5–10 years/11–20 years/More than 20 years
4. Main professional setting: Private individual practice/Multidisciplinary private clinic/Hospital-university clinic/Other
5. Have you attended postgraduate courses or training in periodontology during the last 5 years? Yes, at least one per year/Occasionally/No
6. Are you familiar with the EFP S3 Guidelines for periodontitis treatment? Yes, in detail/Yes, partially/No

#### Appendix A.2.2. Section 2: Perception of Modern Non-Surgical Therapies

7. Standard non-surgical procedures (SRP, supragingival and subgingival scaling) are sufficient for most Stage I–II patients.
8. The use of local antiseptics improves the outcomes of non-surgical treatment.
9. Systemic antibiotic administration (e.g., amoxicillin + metronidazole) should be reserved for severe cases, according to the S3 Guidelines.
10. Modern technologies (e.g., LASER, subgingival air-polishing, Vector, GBT-EMS) are useful in optimizing non-surgical outcomes.
11. The S3 Guidelines emphasize that non-surgical therapy remains the first-line treatment regardless of disease stage.
12. I believe that the effectiveness of non-surgical treatment is minimal without a high level of patient compliance.
13. Rigorous application of Phase 2 of the S3 Guidelines (complex subgingival instrumentation) leads to significant clinical improvement even in Stage III disease.

#### Appendix A.2.3. Section 3: Perception of Surgical Therapy

14. Surgical intervention is essential in the treatment of vertical bone defects or deep periodontal pockets.
15. Periodontal surgery should only be applied after failure of non-surgical treatment, according to the S3 Guidelines.
16. I prefer minimally invasive approaches in selected and eligible cases.
17. I believe that regenerative surgical interventions provide additional long-term advantages compared with non-surgical therapy in Stage III–IV disease.
18. Periodontal surgical therapy allows better visualization and control of areas inaccessible to non-surgical therapy.
19. Surgical procedures have a higher clinical success rate in advanced periodontal disease cases but require careful and objective patient selection.

#### Appendix A.2.4. Section 4: Comparison Between Non-Surgical and Surgical Approaches

20. Non-surgical treatment is better accepted by patients compared with surgical treatment.
21. Clinical outcomes are comparable between non-surgical and surgical therapy in cases of moderate periodontitis, according to the S3 Guidelines.
22. In clinical practice, the surgical approach is sometimes used earlier than recommended by the S3 Guidelines.
23. Non-surgical therapy combined with rigorous reevaluation and periodic professional mechanical plaque removal can prevent the need for surgical intervention.
24. In complex cases, the staged integration of both approaches (non-surgical + surgical) provides the best outcomes.

## Appendix B

**Table A1:** Evaluation of the Impact of Individual Items on Cronbach’s Alpha Coefficient.

Item	Scale Mean if Item Deleted	Scale Variance if Item Deleted	Corrected Item–Total Correlation	Cronbach’s Alpha if Item Deleted
7. Standard non-surgical procedures (SRP, supragingival and subgingival scaling) are sufficient for most Stage I–II periodontal patients.	24.11	12.88	0.49	0.85
8. The use of local antiseptics improves the outcomes of non-surgical treatment.	23.91	11.92	0.67	0.83
9. Systemic antibiotic administration (e.g., amoxicillin + metronidazole) should be reserved for severe cases, according to the S3 Guidelines.	24.11	11.96	0.67	0.83
10. Modern technologies (e.g., LASER, subgingival air-polishing, Vector, GBT-EMS) are useful in optimizing non-surgical outcomes.	23.98	11.75	0.75	0.82
11. The S3 Guidelines emphasize that non-surgical therapy remains the first-line treatment regardless of disease stage.	23.89	11.62	0.68	0.83
12. I consider that the effectiveness of non-surgical treatment is minimal without a high level of patient compliance.	23.98	10.92	0.51	0.87
13. Rigorous application of Phase 2 of the S3 Guidelines (complex subgingival instrumentation) leads to significant clinical improvement even in Stage III disease.	24.04	11.89	0.74	0.82

**Table A2:** Integrated Psychometric Evaluation of the Scale: Reliability and Discriminatory Capacity (ANOVA Test).

		Sum of Squares	df	Mean Square	F	Sig
Between People		243.28	108	2.25		
Within People	Between Items	4.98	6	0.83	2.62	0.01
	Residual	204.73	648	0.31		
	Total	209.71	654	0.32		
Total		452.99	762	0.59		

Grand Mean = 4.0013.

**Table A3:** Intraclass Correlation Coefficient (ICC) Analysis for the Scale Evaluating the Perception of Modern Non-Surgical Periodontal Therapies.

	Intraclass Correlation(a)	95% Confidence Interval		F Test with True Value 0			
		Lower Bound	Upper Bound	Value	df1	df2	Sig
Single Measures	0.46(b)	0.38	0.55	7.13	108	648	0
Average Measures	0.86	0.81	0.89	7.13	108	648	0

**Table A4:** Analysis of Item–Total Correlations for the Scale Evaluating Perceptions of Surgical Periodontal Therapy.

Item	Scale Mean if Item Deleted	Scale Variance if Item Deleted	Corrected Item–Total Correlation	Cronbach’s Alpha if Item Deleted
14. Surgical intervention is essential in the treatment of vertical bone defects or deep periodontal pockets.	20.56	7.28	0.65	0.72
15. Periodontal surgery should be performed only after failure of non-surgical treatment, according to the S3 Guidelines.	20.71	7.39	0.36	0.82
16. I prefer minimally invasive approaches in selected and eligible cases.	20.38	7.48	0.69	0.72
18. I consider that regenerative surgical interventions provide additional long-term advantages compared with non-surgical therapy in Stage III–IV disease.	20.63	7.95	0.44	0.77
19. Periodontal surgical therapy allows better visual access and control of areas inaccessible to non-surgical therapy.	20.38	7.38	0.68	0.72
20. Surgical procedures have a higher clinical success rate in advanced periodontal disease cases, but require careful and objective patient selection.	20.38	7.77	0.53	0.75

**Table A5:** Intraclass Correlation Coefficient (ICC) Analysis—Surgical Therapy Section.

	Intraclass Correlation(a)	95% Confidence Interval		F Test with True Value 0			
		Lower Bound	Upper Bound	Value	df1	df2	Sig
Single Measures	0.38(b)	0.3	0.47	4.72	108	540	0
Average Measures	0.78	0.72	0.84	4.72	108	540	0

**Table A6:** Internal Consistency of the Comparative Scale between Non-Surgical and Surgical Approaches.

Cronbach’s Alpha	N of Items
0.77	5

**Table A7:** ANOVA Analysis of Differences between Items—Comparative Section between Non-Surgical and Surgical Approaches.

		Sum of Squares	df	Mean Square	F	Sig
Between People		158.08	108	1.46		
Within People	Between Items	50.40	4	12.60	38.12	0
	Residual	142.79	432	0.33		
	Total	193.2	436	0.44		
Total		351.28	544	0.64		

**Table A8:** Intraclass Correlation Coefficient (ICC)—Comparative Section between Non-Surgical and Surgical Approaches.

	Intraclass Correlation(a)	95% Confidence Interval		F Test with True Value 0			
		Lower Bound	Upper Bound	Value	df1	df2	Sig
Single Measures	0.40(b)	0.31	0.50	4.42	108	432	0
Average Measures	0.77	0.69	0.83	4.42	108	432	0

**Table A9:** KMO and Bartlett’s Test—Data Adequacy for Factor Analysis.

Kaiser-Meyer-Olkin Measure of Sampling Adequacy		0.77
Bartlett’s Test of Sphericity	Approx. Chi-Square	693.63
	df	78
	Sig.	0
